# Mendelian randomization study on the causal relationship between food and cholelithiasis

**DOI:** 10.3389/fnut.2024.1276497

**Published:** 2024-03-04

**Authors:** Zhicheng Liu, Shun Liu, Peizhe Song, Yan Jiao

**Affiliations:** ^1^Department of Gastric and Intestinal, General Surgery Center, First Hospital of Jilin University, Changchun, China; ^2^Department of Hepatobiliary and Pancreatic Surgery, General Surgery Center, First Hospital of Jilin University, Changchun, China

**Keywords:** food, cholelithiasis, causal relationship, Mendelian randomization, risk factor

## Abstract

**Background:**

Cholelithiasis, commonly referred to as gallstones, is a prevalent medical condition influenced by a combination of genetic factors, lifestyle choices, and dietary habits. Specific food items have been associated with an increased susceptibility to cholelithiasis, whereas others seem to offer a protective effect against its development.

**Methods:**

In this study, we conducted a Mendelian randomization (MR) analysis using a large-scale genetic dataset comprising individuals with European ancestry to explore the potential causal relationship between diet and cholelithiasis. The analysis incorporated 17 food-related variables, which were considered as potential factors influencing the occurrence of this condition.

**Results:**

Our findings indicate that a higher consumption of cooked vegetables, dried fruit, and oily fish is associated with a reduced risk of cholelithiasis. Conversely, a higher consumption of lamb is associated with an increased risk of developing the condition. Importantly, these associations proved robust to sensitivity and heterogeneity tests, and the pleiotropic test results further supported the hypothesis of a causal relationship between diet and cholelithiasis.

**Conclusion:**

Through our study, we provide compelling evidence for the existence of a causal relationship between diet and cholelithiasis. Adopting a dietary pattern enriched with cooked vegetables, dried fruit, and oily fish, while minimizing lamb intake, may contribute to the prevention of cholelithiasis. Recognizing diet as a modifiable risk factor in the prevention and management of this condition is of paramount importance, and our study offers valuable insights in this regard.

## 1 Introduction

Cholelithiasis, commonly referred to as gallstones, is a widespread medical condition affecting a substantial global population (1). The development of gallstones involves a multifactorial process, influenced by genetic factors, lifestyle choices, and dietary patterns (2–5). Over recent decades, the prevalence of cholelithiasis has been on the rise, affecting approximately 10–15% of adults in industrialized nations (6). Related risk factors have aroused public concern. These include supersaturation of cholesterol, hypomotility of the gallbladder, excess bilirubin, age, ethnicity, female gender, disease history, family history, smoking, alcohol consumption, body mass index (BMI), and cholesterol levels (7). Additionally, bariatric surgery, a treatment for obesity, can also increase the risk of cholelithiasis, with Caucasian race and female sex being identified as risk factors (8). Gilbert syndrome, a hereditary hyperbilirubinemia, has been associated with an increased risk of cholelithiasis in children, although it is not the sole risk factor (9). However, the relationship between risk factors and cholelithiasis is not explained clearly.

Dietary factors have been identified as significant contributors to the risk of cholelithiasis, with certain foods associated with an increased likelihood of gallstone formation. High consumption of agricultural products, fat, and sugar has been linked to elevated risk, while a diet rich in fiber, fruits, and vegetables appears to have a protective effect (10). However, the causal nature of the relationship between food intake and cholelithiasis remains uncertain, necessitating further investigation to ascertain the impact of diet on this condition.

Mendelian randomization (MR) serves as a valuable tool for exploring causality in observational studies. By utilizing genetic variants as proxies for exposures, MR offers a more robust assessment of the causal relationship between an exposure, such as dietary factors, and an outcome, like cholelithiasis, as it mitigates the influence of confounding and reverse causality (11).

This study aims to investigate the causal connection between specific food items and cholelithiasis through MR analysis, encompassing variables such as alcohol intake frequency, various food types (beef, bread, cereal, cheese, coffee, cooked vegetables, dried fruit, fresh fruit, lamb/mutton, non-oily fish, oily fish, pork, poultry, processed meat, salad/raw vegetables, and tea). The findings provide a comprehensive evaluation of the causal link between diet and cholelithiasis, underscoring the significance of considering diet as a modifiable risk factor in both the prevention and management of this condition.

## 2 Methods

### 2.1 Study design

This study aims to elucidate the causal relationships between 17 common food exposures and the development of cholelithiasis (gallstones) by employing a robust two-pronged methodological approach: two-sample Mendelian randomization (MR) analysis and multivariable MR (MVMR) analysis. By leveraging genetic variants as instrumental variables (IVs) for food exposures, these methods allow us to infer causality from observational data while minimizing confounding factors and reverse causation, common limitations in traditional observational studies.

The genetic proxies for the 17 food exposures were Identified using data from the GIANT (Genetic Investigation of ANThropometric Traits) consortium, which provides a comprehensive resource for understanding genetic influences on dietary habits.

The incidence of cholelithiasis, specifically calculus of the gallbladder without cholecystitis, was assessed using data from the UK Biobank, a large-scale biomedical database and research resource containing in-depth genetic and health information from half a million UK participants.

### 2.2 Two-sample MR analysis

The two-sample MR analysis aimed to estimate the causal effect of each food exposure on cholelithiasis (12). The remaining SNPs’ power was then evaluated using F statistics (F = beta^2^/se^2^) for each SNP, and an overall F statistic was generated for all SNPs. This analysis employed three methods: the Inverse Variance Weighted (IVW) method, MR-Egger regression, and the weighted median approach. The IVW method served as the primary approach, while MR-Egger regression was employed to control for pleiotropy effects. The weighted median approach combined results from multiple MR estimates to estimate the causal effect of each food exposure.

### 2.3 Multivariable MR analysis

The MVMR analysis sought to explore the causal relationship between multiple food exposures and cholelithiasis using the MVMR package (13). This analysis controlled for potential confounding effects arising from multiple exposures, enabling the estimation of the causal effect of each food exposure on cholelithiasis.

### 2.4 Sensitivity tests

To assess the robustness of the results, sensitivity tests were performed, and individuals with missing data were excluded to mitigate potential confounding effects.

### 2.5 Heterogeneity test

A heterogeneity test was conducted to determine the presence of between-study heterogeneity in the results. The Cochran’s Q statistic and the I2 statistic were used for this purpose. The Cochran’s Q statistic tested for heterogeneity between studies, while the I2 statistic quantified the proportion of total variation attributable to between-study heterogeneity.

### 2.6 Pleiotropic test

A pleiotropic test was performed to investigate the presence of pleiotropy, wherein a single genetic variant influences multiple traits. The MR-PRESSO method, a gene-based test combining multiple MR estimates, was employed for detecting pleiotropy.

### 2.7 Data analysis

Data analysis was carried out using the R statistical software. The two-sample MR analysis utilized the TwosampleMR packages, while the MVMR analysis employed the MVMR package. Sensitivity tests, heterogeneity tests, and pleiotropic tests were conducted using the MR-PRESSO package. All statistical tests were two-tailed and performed at a significance level of 0.05.

## 3 Results

**Figure 1:**
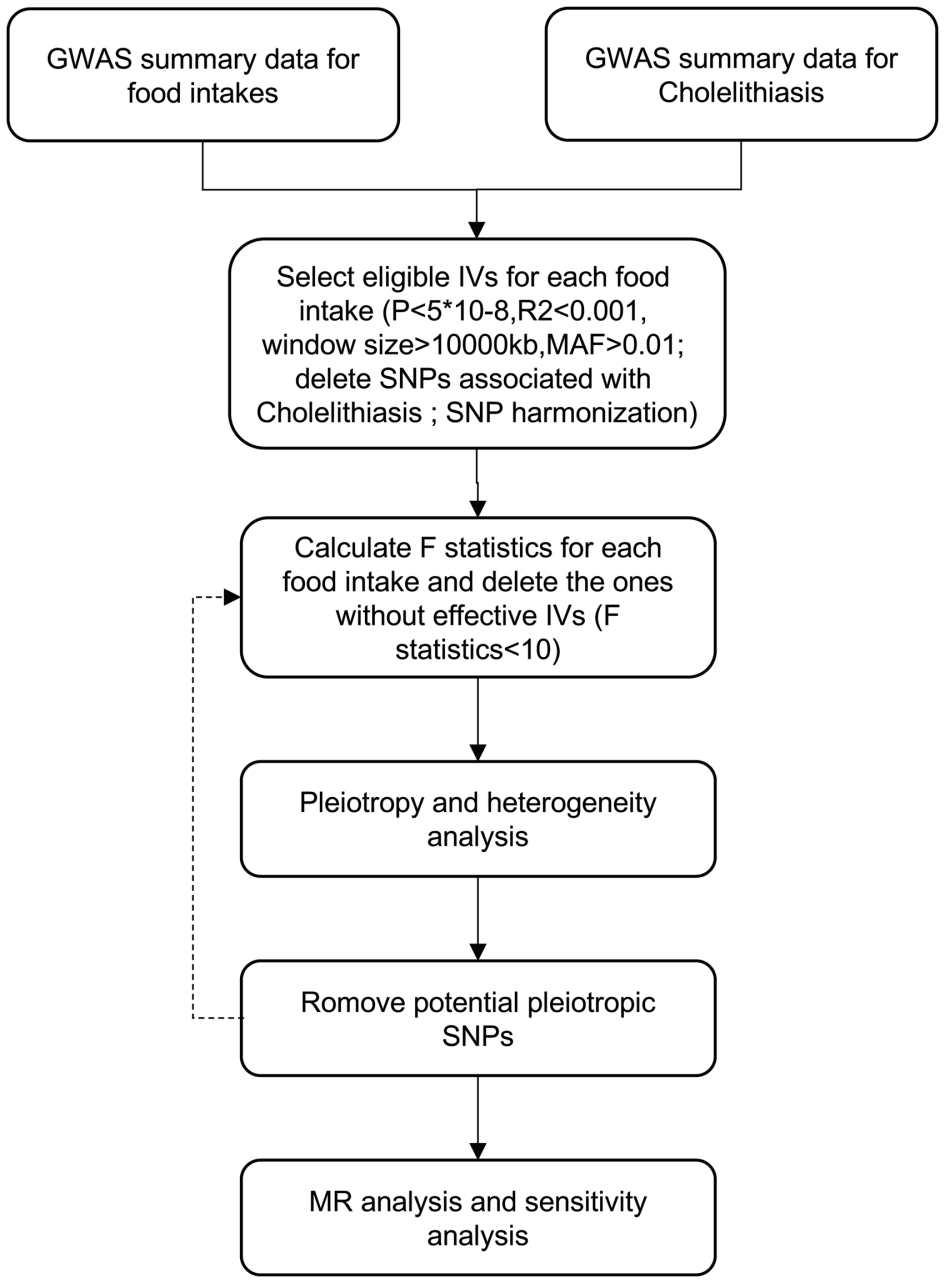
Flowchart of MR analysis in this study.

Cholelithiasis, also known as gallstone disease, is a prevalent condition affecting a considerable global population. Although the precise etiology of cholelithiasis remains incompletely understood, lifestyle factors, particularly dietary habits, are believed to contribute significantly. In this study, we employed Mendelian randomization (MR) analysis to investigate the potential causal relationship between various food exposures and cholelithiasis. The study design is represented in Figure 1.

### 3.1 Overview of data source

Data from the Global Assessment of Well-Being Study (GAWS) were analyzed, encompassing variables related to alcohol intake frequency, as well as the consumption of specific food items, such as beef, bread, cereal, cheese, coffee, cooked vegetables, dried fruit, fresh fruit, lamb/mutton, non-oily fish, oily fish, pork, poultry, processed meat, salad/raw vegetables, and tea (Table 1). The sample size was substantial, comprising over 400,000 individuals of European descent.

**Table 1:** GWAS information of food involved in this study.

Trait	Population	Sex	Unit	Year	Sample_size	NSNP	Consortium	Author
Alcohol intake frequency	European	Males and females	SD	2017	336,965	10,894,596	Neale Lab	Neale
Beef intake	European	Males and females	SD	2018	461,053	9,851,867	MRC-IEU	Ben Elsworth
Bread intake	European	Males and females	SD	2018	452,236	9,851,867	MRC-IEU	Ben Elsworth
Cereal intake	European	Males and females	SD	2018	441,640	9,851,867	MRC-IEU	Ben Elsworth
Cheese intake	European	Males and females	SD	2018	451,486	9,851,867	MRC-IEU	Ben Elsworth
Coffee intake	European	Males and females	SD	2018	428,860	9,851,867	MRC-IEU	Ben Elsworth
Cooked vegetable intake	European	Males and females	SD	2018	448,651	9,851,867	MRC-IEU	Ben Elsworth
Dried fruit intake	European	Males and females	SD	2018	421,764	9,851,867	MRC-IEU	Ben Elsworth
Fresh fruit intake	European	Males and females	SD	2018	446,462	9,851,867	MRC-IEU	Ben Elsworth
Lamb/mutton intake	European	Males and females	SD	2018	460,006	9,851,867	MRC-IEU	Ben Elsworth
Non-oily fish intake	European	Males and females	SD	2018	460,880	9,851,867	MRC-IEU	Ben Elsworth
Oily fish intake	European	Males and females	SD	2018	460,443	9,851,867	MRC-IEU	Ben Elsworth
Pork intake	European	Males and females	SD	2018	460,162	9,851,867	MRC-IEU	Ben Elsworth
Poultry intake	European	Males and females	SD	2018	461,900	9,851,867	MRC-IEU	Ben Elsworth
Processed meat intake	European	Males and females	SD	2018	461,981	9,851,867	MRC-IEU	Ben Elsworth
Salad / raw vegetable intake	European	Males and females	SD	2018	435,435	9,851,867	MRC-IEU	Ben Elsworth
Tea intake	European	Males and females	SD	2018	447,485	9,851,867	MRC-IEU	Ben Elsworth

### 3.2 Two-sample MR analysis

**Figure 2:**
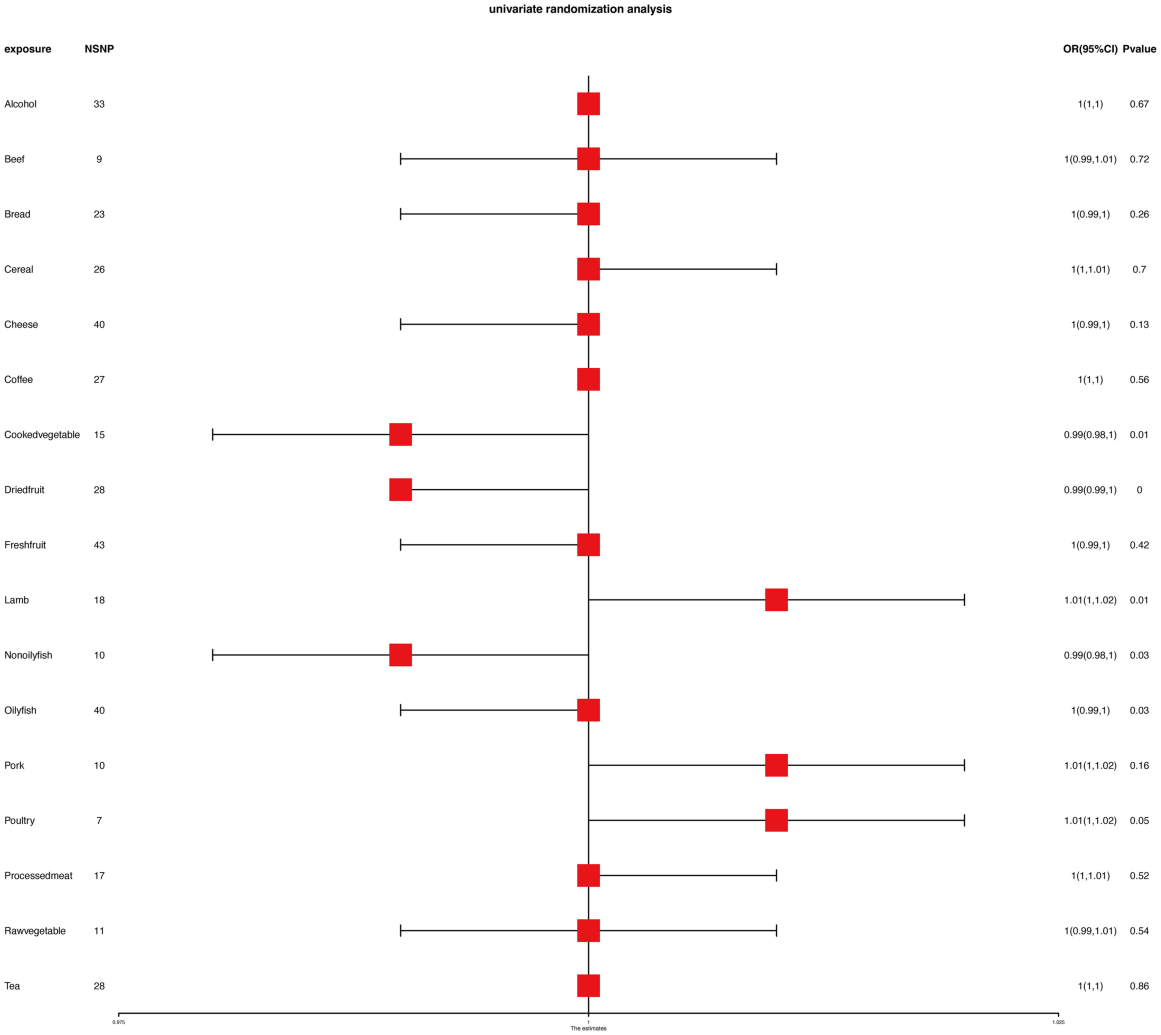
MR analysis results of food on cholelithiasis.

**Figure 3:**
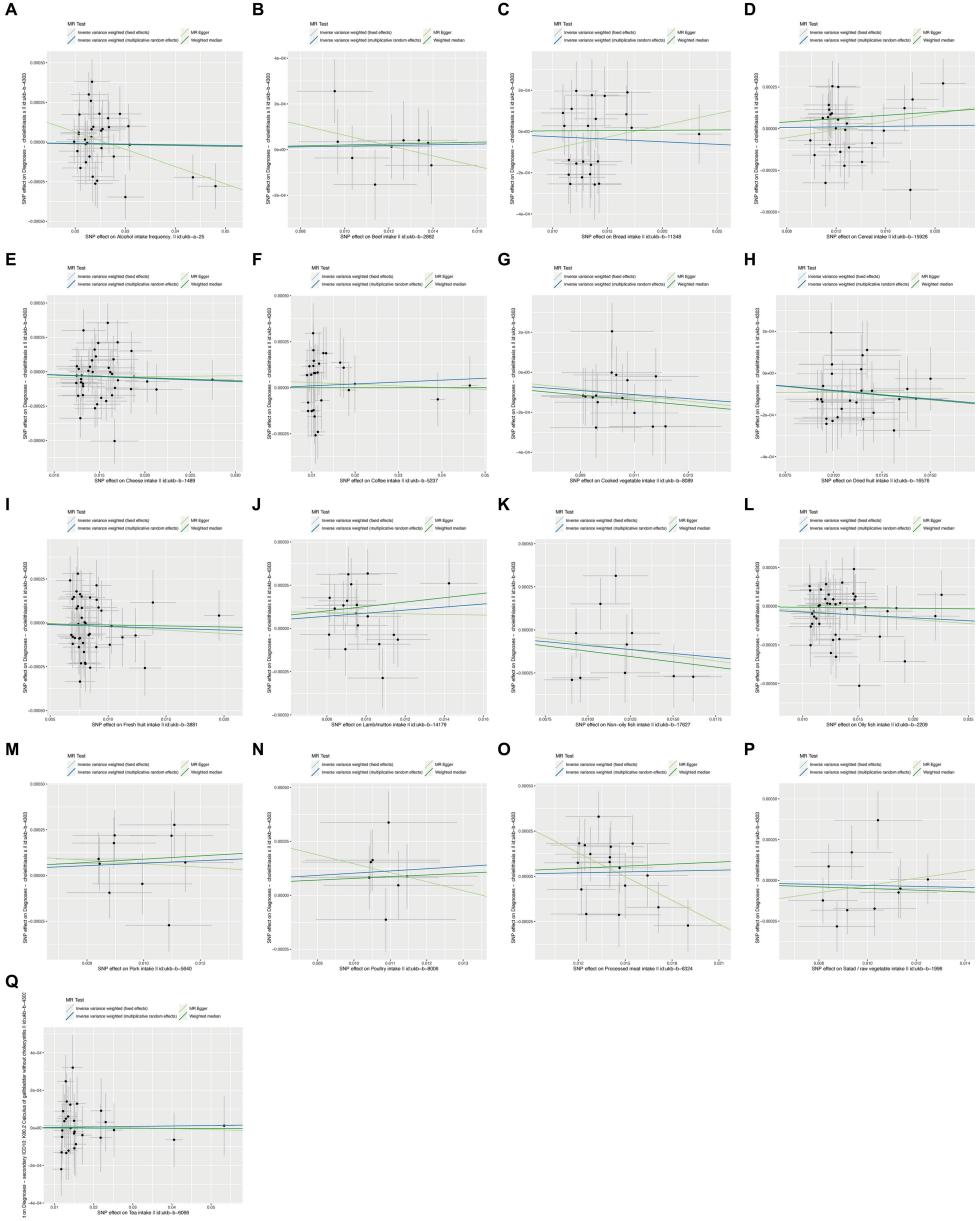
Scatter plots of food on cholelithiasis. **(A)** Alcohol; **(B)** Beef; **(C)** Bread; **(D)** Cereal; **(E)** Cheese; **(F)** Coffee; **(G)** Cooked vegetable; **(H)** Dried fruit; **(I)** Fresh fruit; **(J)** Lamb/mutton; **(K)** Non-oily fish; **(L)** Oily fish; **(M)** Pork; **(N)** Poultry; **(O)** Processed meat; **(P)** Salad/raw vegetable; **(Q)** Tea.

**Figure 4:**
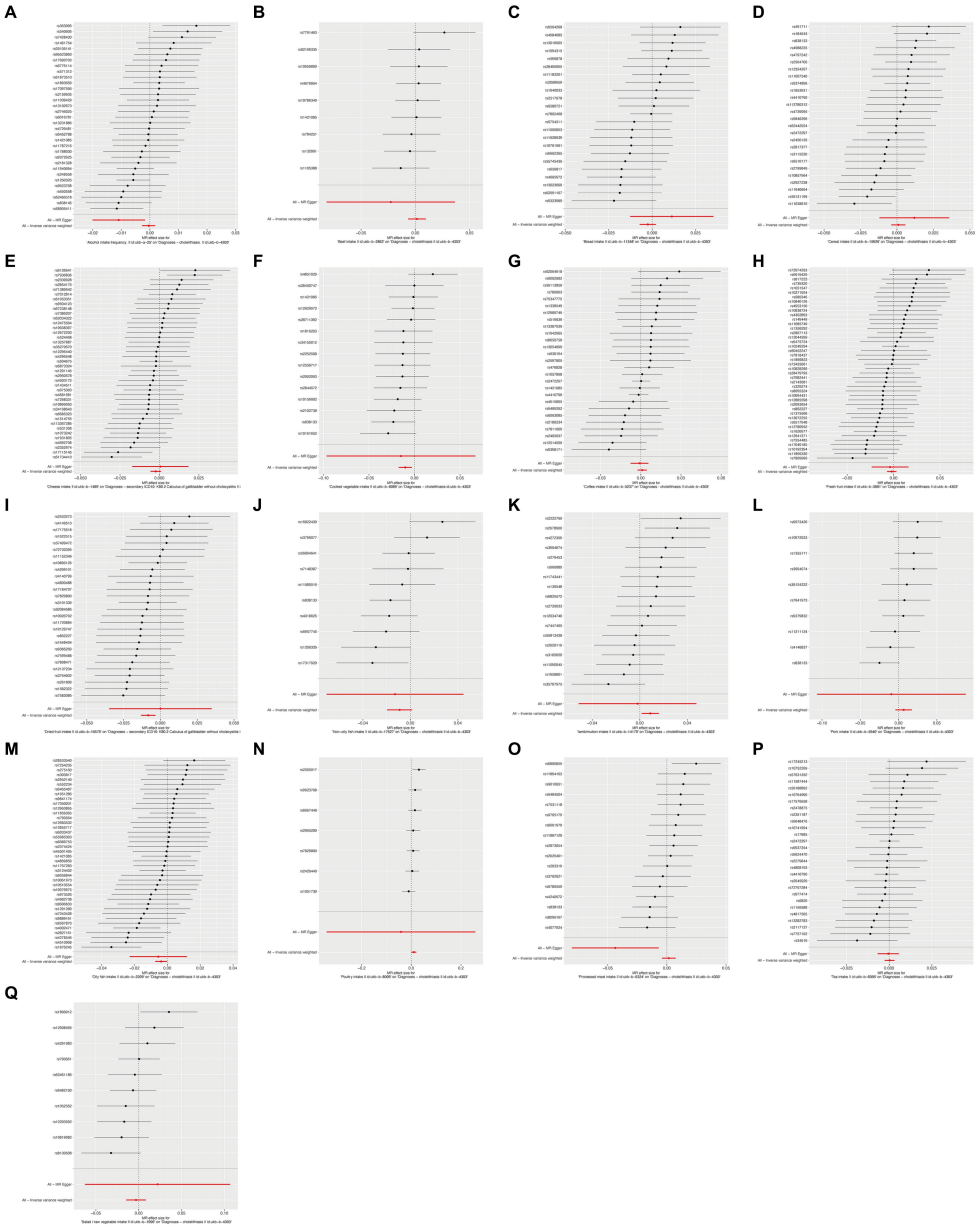
Forest plots of food on cholelithiasis. **(A)** Alcohol; **(B)** Beef; **(C)** Bread; **(D)** Cereal; **(E)** Cheese; **(F)** Cooked vegetable; **(G)** Coffee; **(H)** Fresh fruit; **(I)** Dried fruit; **(J)** Non-oily fish; **(K)** Lamb/mutton; **(L)** Pork; **(M)** Oily fish; **(N)** Poultry; **(O)** Processed meat; **(P)** Tea; **(Q)** Salad/raw vegetable.

**Figure 5:**
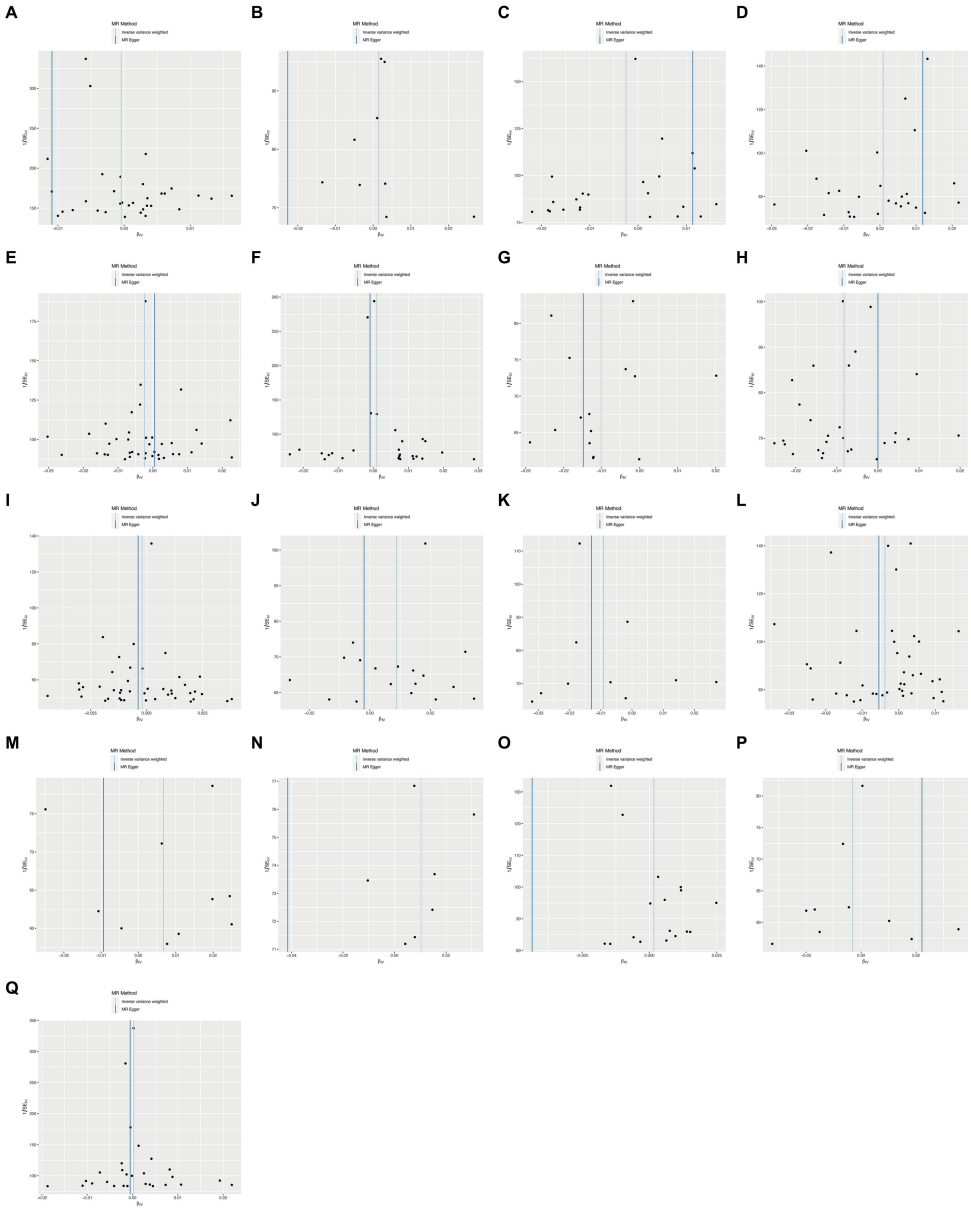
Funnel plots of food on cholelithiasis. **(A)** Alcohol; **(B)** Beef; **(C)** Bread; **(D)** Cereal; **(E)** Cheese; **(F)** Coffee; **(G)** Cooked vegetable; **(H)** Dried fruit; **(I)** Fresh fruit; **(J)** Lamb/mutton; **(K)** Non-oily fish; **(L)** Oily fish; **(M)** Pork; **(N)** Poultry; **(O)** Processed meat; **(P)** Salad/raw vegetable; **(Q)** Tea.

**Figure 6:**
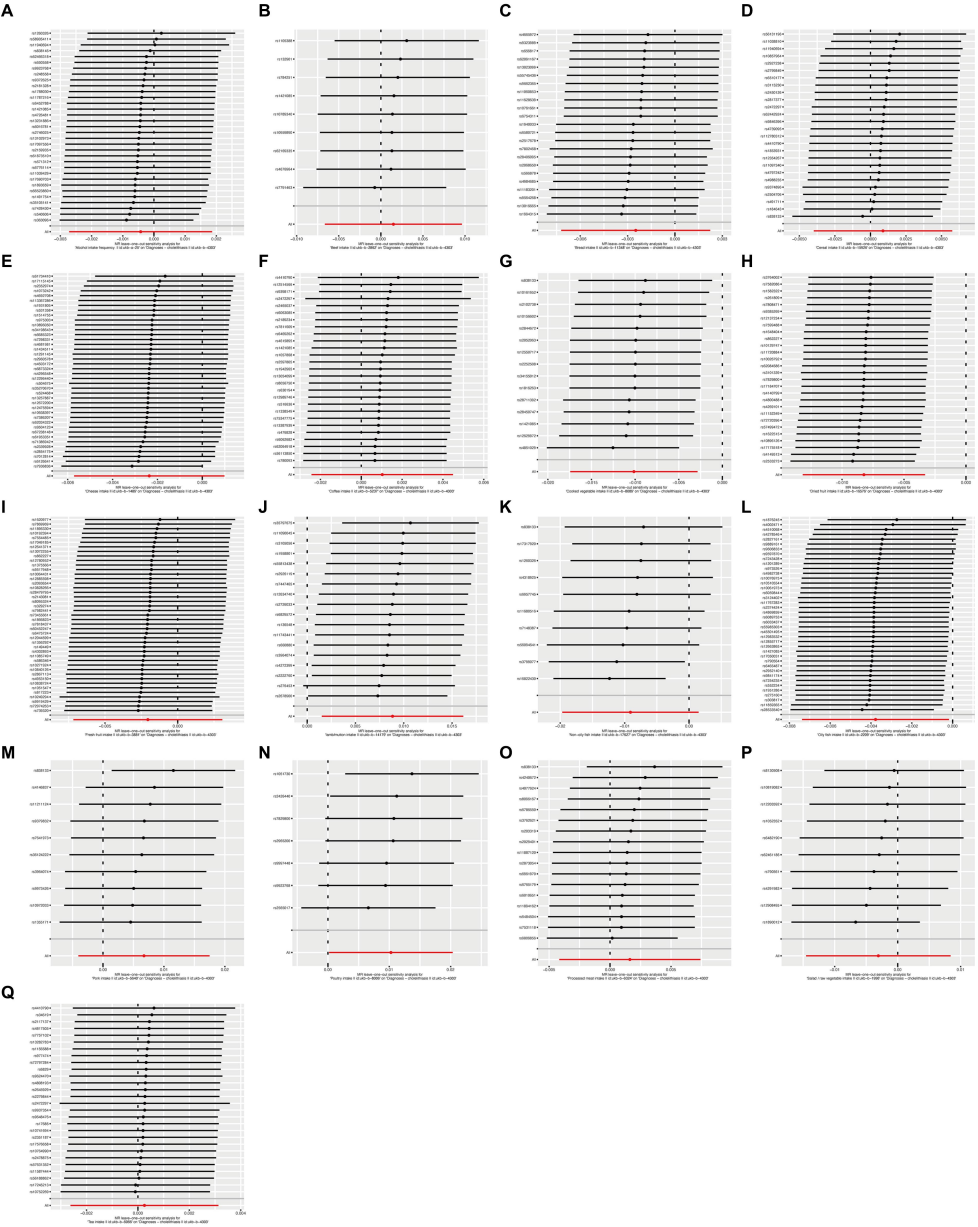
Leave-one-out plots of food on cholelithiasis. **(A)** Alcohol; **(B)** Beef; **(C)** Bread; **(D)** Cereal; **(E)** Cheese; **(F)** Coffee; **(G)** Cooked vegetable; **(H)** Dried fruit; **(I)** Fresh fruit; **(J)** Lamb/mutton; **(K)** Non-oily fish; **(L)** Oily fish; **(M)** Pork; **(N)** Poultry; **(O)** Processed meat; **(P)** Salad/raw vegetable; **(Q)** Tea.

To ensure the robustness of our findings, we performed sensitivity tests, heterogeneity tests, and pleiotropic tests. As depicted in Figure 2, our analysis identified statistically significant causal relationships between cooked vegetables, dried fruit, lamb, oily fish, and cholelithiasis, as determined through both two-sample MR analysis, the intake of cooked vegetables was associated with a reduced risk of cholelithiasis, with an Odds Ratio (OR) of 0.990 (95% CI 0.983–0.997) and a *p*-value of 0.006. Similarly, dried fruit intake displayed a lower risk of cholelithiasis, with an OR of 0.992 (95% CI 0.987–0.997) and a *p*-value of 0.001. Oily fish consumption was also linked to a decreased risk, with an OR of 0.996 (95% CI 0.993–1.000) and a *p*-value of 0.026. In contrast, the consumption of lamb/mutton was associated with an elevated risk, showing an OR of 1.009 (95% CI 1.002–1.016) and a *p*-value of 0.012. Likewise, poultry intake displayed a higher risk, with an OR of 1.010 (95% CI 1.000–1.021) and a *p*-value of 0.046. Additional methods, including MR-Egger, weighted median, and IVW mre, providing additional support for our findings, are presented in Table 2. Figures 3A–Q, 4A–Q, 5A–Q, 6A–Q showcase the scatter plots, forest plots, funnel plots, and leave-one-out plots for cholelithiasis, respectively.

**Table 2:** MR analysis of food intakes on cholelithiasis in complementary methods.

Exposure	NSNP	MR_methodology	OR	LCI95	UCI95	*P*-value	*Q*-value	Pheterogeneity	MR_Egger_intercept	Ppeleiotropy	Rsquare	Fstat
Alcohol	33	IVW_fe	1.000	0.998	1.002	0.665	45.182	0.061			0.103	59.419
		MR_Egger	0.989	0.980	0.998	0.027	38.613	0.163	0.000	0.029		
		Weighted_median	0.999	0.996	1.003	0.731						
		IVW_mre	1.000	0.997	1.002	0.716	45.182	0.061				
Beef	9	IVW_fe	1.001	0.993	1.010	0.719	4.707	0.788			0.027	41.473
		MR_Egger	0.978	0.921	1.037	0.478	4.055	0.773	0.000	0.446		
		Weighted_median	1.002	0.992	1.012	0.704						
		IVW_mre	1.001	0.995	1.008	0.639	4.707	0.788				
Bread	23	IVW_fe	0.998	0.993	1.002	0.261	26.080	0.248			0.091	41.814
		MR_Egger	1.011	0.987	1.036	0.365	24.529	0.268	0.000	0.262		
		Weighted_median	1.000	0.994	1.007	0.929						
		IVW_mre	0.998	0.993	1.002	0.302	26.080	0.248				
Cereal	26	IVW_fe	1.001	0.996	1.005	0.695	30.270	0.214			0.002	45.030
		MR_Egger	1.012	0.988	1.036	0.340	29.238	0.211	0.000	0.366		
		Weighted_median	1.005	0.999	1.012	0.129						
		IVW_mre	1.001	0.996	1.006	0.722	30.270	0.214				
Cheese	40	IVW_fe	0.998	0.995	1.001	0.127	47.522	0.164			0.099	39.017
		MR_Egger	1.001	0.983	1.019	0.952	47.388	0.141	0.000	0.746		
		Weighted_median	0.998	0.993	1.002	0.352						
		IVW_mre	0.998	0.994	1.001	0.166	47.522	0.164				
Coffee	27	IVW_fe	1.001	0.998	1.005	0.561	23.107	0.627			0.185	72.712
		MR_Egger	0.999	0.992	1.006	0.785	22.646	0.598	0.000	0.503		
		Weighted_median	1.000	0.995	1.005	0.999						
		IVW_mre	1.001	0.998	1.004	0.538	23.107	0.627				
CookedVegetable	15	IVW_fe	0.990	0.983	0.997	0.006	10.212	0.747			0.032	37.584
		MR_Egger	0.985	0.908	1.069	0.728	10.199	0.678	0.000	0.911		
		Weighted_median	0.987	0.977	0.998	0.015						
		IVW_mre	0.990	0.984	0.996	0.001	10.212	0.747				
DriedFruit	28	IVW_fe	0.992	0.987	0.997	0.001	17.286	0.924			0.075	41.899
		MR_Egger	1.000	0.966	1.035	0.999	17.066	0.907	0.000	0.643		
		Weighted_median	0.992	0.985	0.998	0.016						
		IVW_mre	0.992	0.988	0.996	0.000	17.286	0.924				
FreshFruit	43	IVW_fe	0.998	0.993	1.003	0.420	43.998	0.387			0.040	45.950
		MR_Egger	0.996	0.977	1.016	0.709	43.963	0.347	0.000	0.858		
		Weighted_median	0.999	0.991	1.007	0.767						
		IVW_mre	0.998	0.993	1.003	0.431	43.998	0.387				
Lamb	18	IVW_fe	1.009	1.002	1.016	0.012	19.172	0.319			0.077	39.595
		MR_Egger	0.998	0.950	1.049	0.941	18.953	0.271	0.000	0.673		
		Weighted_median	1.013	1.002	1.023	0.015						
		IVW_mre	1.009	1.002	1.016	0.018	19.172	0.319				
NonOilyFish	10	IVW_fe	0.991	0.983	0.999	0.026	15.761	0.072			0.006	44.802
		MR_Egger	0.987	0.932	1.046	0.672	15.726	0.046	0.000	0.897		
		Weighted_median	0.988	0.976	0.999	0.039						
		IVW_mre	0.991	0.981	1.001	0.092	15.761	0.072				
OilyFish	40	IVW_fe	0.996	0.993	1.000	0.026	45.442	0.221			0.029	44.918
		MR_Egger	0.995	0.977	1.012	0.545	45.398	0.191	0.000	0.850		
		Weighted_median	0.999	0.994	1.004	0.792						
		IVW_mre	0.996	0.993	1.000	0.040	45.442	0.221				
Pork	10	IVW_fe	1.007	0.997	1.016	0.162	11.700	0.231			0.015	37.686
		MR_Egger	0.991	0.899	1.092	0.854	11.546	0.173	0.000	0.752		
		Weighted_median	1.009	0.996	1.022	0.162						
		IVW_mre	1.007	0.996	1.018	0.220	11.700	0.231				
Poultry	7	IVW_fe	1.010	1.000	1.021	0.046	5.239	0.514			0.009	32.539
		MR_Egger	0.959	0.711	1.294	0.797	5.121	0.401	0.001	0.749		
		Weighted_median	1.008	0.994	1.022	0.283						
		IVW_mre	1.010	1.001	1.020	0.032	5.239	0.514				
ProcessedMeat	17	IVW_fe	1.002	0.997	1.007	0.524	21.408	0.163			0.036	38.536
		MR_Egger	0.957	0.923	0.993	0.035	15.351	0.426	0.001	0.028		
		Weighted_median	1.004	0.996	1.011	0.327						
		IVW_mre	1.002	0.996	1.007	0.582	21.408	0.163				
RawVegetable	11	IVW_fe	0.997	0.987	1.007	0.537	12.480	0.188			0.023	38.333
		MR_Egger	1.022	0.939	1.113	0.623	11.963	0.153	0.000	0.573		
		Weighted_median	0.995	0.982	1.008	0.462						
		IVW_mre	0.997	0.986	1.008	0.600	12.480	0.188				
Tea	28	IVW_fe	1.000	0.997	1.003	0.862	16.259	0.948			0.067	60.812
		MR_Egger	0.999	0.993	1.006	0.871	16.183	0.932	0.000	0.785		
		Weighted_median	1.000	0.996	1.004	0.976						
		IVW_mre	1.000	0.998	1.002	0.823	16.259	0.948				

### 3.3 MVMR analysis

**Figure 7:**
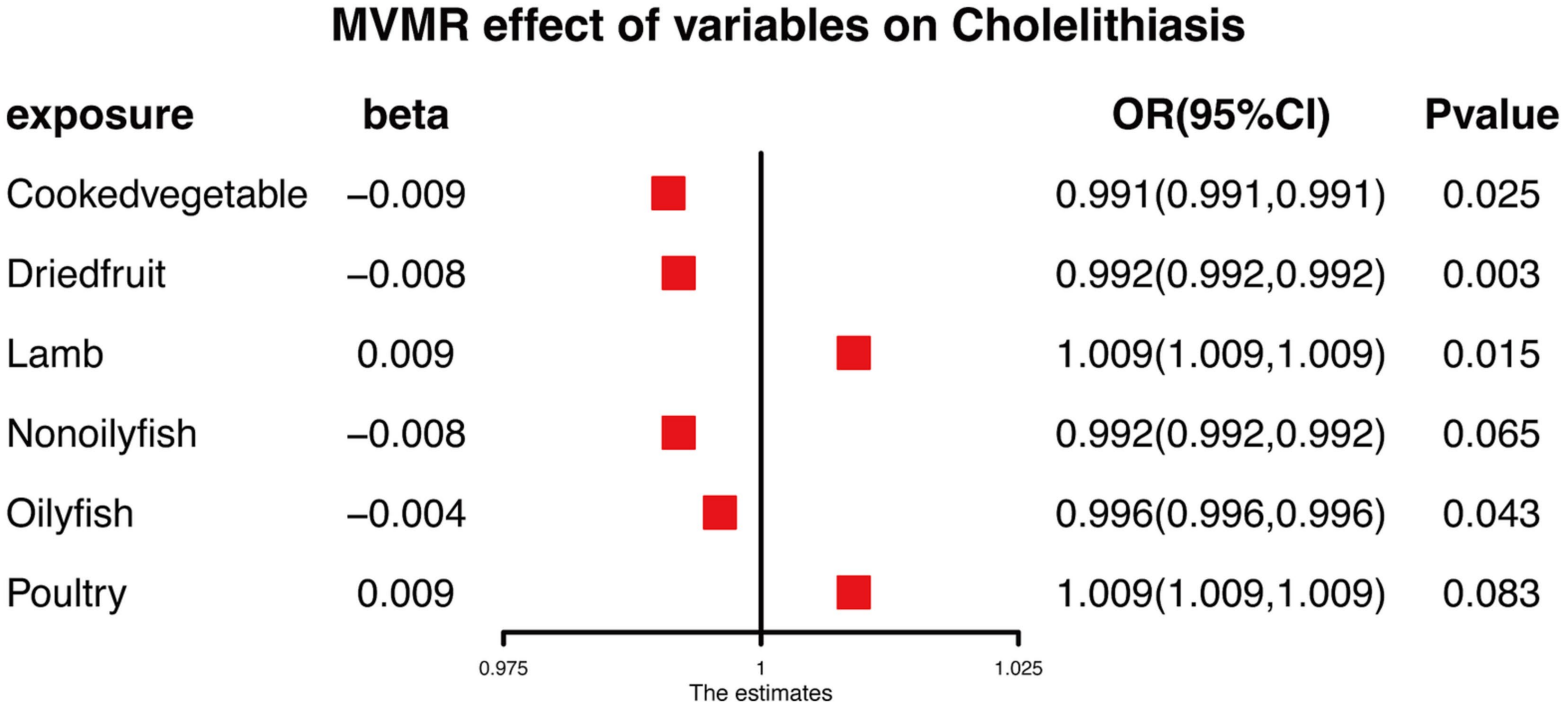
MVMR analysis results of food on cholelithiasis.

Ultimately, our analysis identified statistically significant causal relationships between cooked vegetables, dried fruit, lamb, oily fish, and cholelithiasis, as determined through multivariable MR (MVMR) analysis (14). The harmonized data in the MVMR analysis was shown in Supplementary Table S1. Furthermore, multivariable MR analysis confirmed that Cooked Vegetable, Dried Fruit, and Oily Fish were significantly associated with a reduced risk of cholelithiasis, while lamb exhibited an increased risk (Figure 7).

### 3.4 Reverse causation analysis

In order to reduce the reverse causation, we did a reverse mendelian randomization analysis to explore the effects of cholelithiasis on food intake. However, the results provided some evidence that gallstone disease may influence lamb and oily fish intake (Supplementary Figure S1).

Overall, our results suggest the existence of potential causal relationships between certain food exposures and the risk of cholelithiasis. However, further investigations are warranted to validate these findings and explore the underlying mechanisms. Nonetheless, these insights shed valuable light on the role of diet in the development of cholelithiasis and may hold implications for the prevention and management of this prevalent condition.

## 4 Discussion

The findings of this study indicate a positive causal association between cholelithiasis and the consumption of cooked vegetables, dried fruits, lamb/mutton, and oily fish. The results remained robust when subjected to sensitivity tests, exhibiting low between-study heterogeneity and no evidence of pleiotropy. These observations are consistent with previous observational studies that have linked higher consumption of these food items with an increased risk of cholelithiasis (14, 15).

In comparison to prior research, previous observational studies have reported an elevated risk of cholelithiasis associated with increased intake of red meat, including beef and pork (16). However, our study did not establish a causal relationship between beef and pork consumption and cholelithiasis. This disparity may be attributed to variations in the preparation and cooking methods of red meat among different populations, potentially affecting its association with cholelithiasis.

Our findings also suggest a potential role of cooked vegetables in the development of cholelithiasis, opposing to previous observational studies indicating an increased risk with high vegetable consumption (17–19). The potential reason might be the difference of study types and ethnic population. Therefore, RCT research with stronger evidence is needed. Importantly, no causal relationship was found between raw vegetable consumption and cholelithiasis, implying that the cooking process may influence the association between vegetables and cholelithiasis. This emphasizes the significance of considering food preparation and cooking methods in dietary research related to health (20, 21).

Moreover, we identified a novel positive causal relationship between dried fruits and cholelithiasis, where higher consumption of dried fruits was associated with an increased risk. The specific mechanism underlying this association remains unclear; however, it may be related to the high sugar and fructose content in dried fruits, potentially elevating bile acids and cholesterol in the bile and contributing to gallstone formation (22, 23).

Additionally, our results indicated that lamb/mutton consumption may contribute to the development of cholelithiasis, consistent with prior observational studies linking high consumption of lamb/mutton to an increased risk (14, 24–26). The mechanism explaining this relationship is not fully understood, but it is suggested that the high fat content in lamb/mutton may raise bile acids and cholesterol levels in the bile, leading to gallstone formation.

Lastly, oily fish consumption was associated with a reduced risk of cholelithiasis, in line with previous observational studies highlighting the protective effect of oily fish consumption (25, 26). The exact mechanism by which oily fish reduces the risk of cholelithiasis remains uncertain, but it may be related to its high fat content, potentially influencing bile acids and cholesterol levels in the bile, thereby reducing gallstone formation.

While this study provides valuable evidence for a causal relationship between certain food exposures and cholelithiasis, several limitations should be acknowledged. Even though most food exposures revealed no reverse causation, lamb and fatty fish did demonstrate potential bidirectional connections with cholelithiasis. In order to properly describe the temporal association between these two dietary exposures and gallstone disease, it was necessary to do more studies utilizing longitudinal data or other methods. The results were based on a limited number of genetic variants, and other unanalyzed variants may also influence the relationship between food exposures and cholelithiasis. Additionally, the study was conducted in a specific ethnic population, limiting the generalizability of the findings to other populations. The potential influence of residual confounding from other factors affecting the relationship between food exposures and cholelithiasis was not fully accounted for. Furthermore, lifestyle factors such as physical activity and smoking, which may impact cholelithiasis development, were not considered (27, 28).

Despite these limitations, this study contributes valuable insights to the existing literature, offering robust evidence for a causal relationship between specific food exposures and cholelithiasis. These findings hold implications for dietary recommendations aimed at cholelithiasis prevention and underscore the importance of considering food preparation and cooking methods in studies exploring the association between diet and health. Further research is warranted to validate these findings across diverse populations and elucidate the underlying mechanisms connecting food exposures to cholelithiasis.

In conclusion, this study provides evidence supporting a positive causal relationship between cholelithiasis and the consumption of cooked vegetables, dried fruits, lamb/mutton, and oily fish. The findings emphasize the significance of considering food preparation and cooking methods in dietary research and may inform future dietary recommendations for cholelithiasis prevention.

## Data availability statement

The raw data supporting the conclusions of this article will be made available by the authors, without undue reservation.

## Author contributions

ZL: Writing – original draft. SL: Methodology, Validation, Supervision, Writing – original draft. PS: Data curation, Formal analysis, Software, Writing – original draft. YJ: Conceptualization, Writing – review & editing.

## Supplementary material

Supplymentary Figure S1 Reverse causation analysis.

Supplymentary Table S1 The harmonized data in the MVMR analysis.
