# Supplementary figures and images for: Mendelian randomization study on the causal relationship between food and cholelithiasis
Source: Front Nutr. 2024 Mar 4;11:1276497. doi: 10.3389/fnut.2024.1276497 (PMC10944874; doi:10.3389/fnut.2024.1276497)

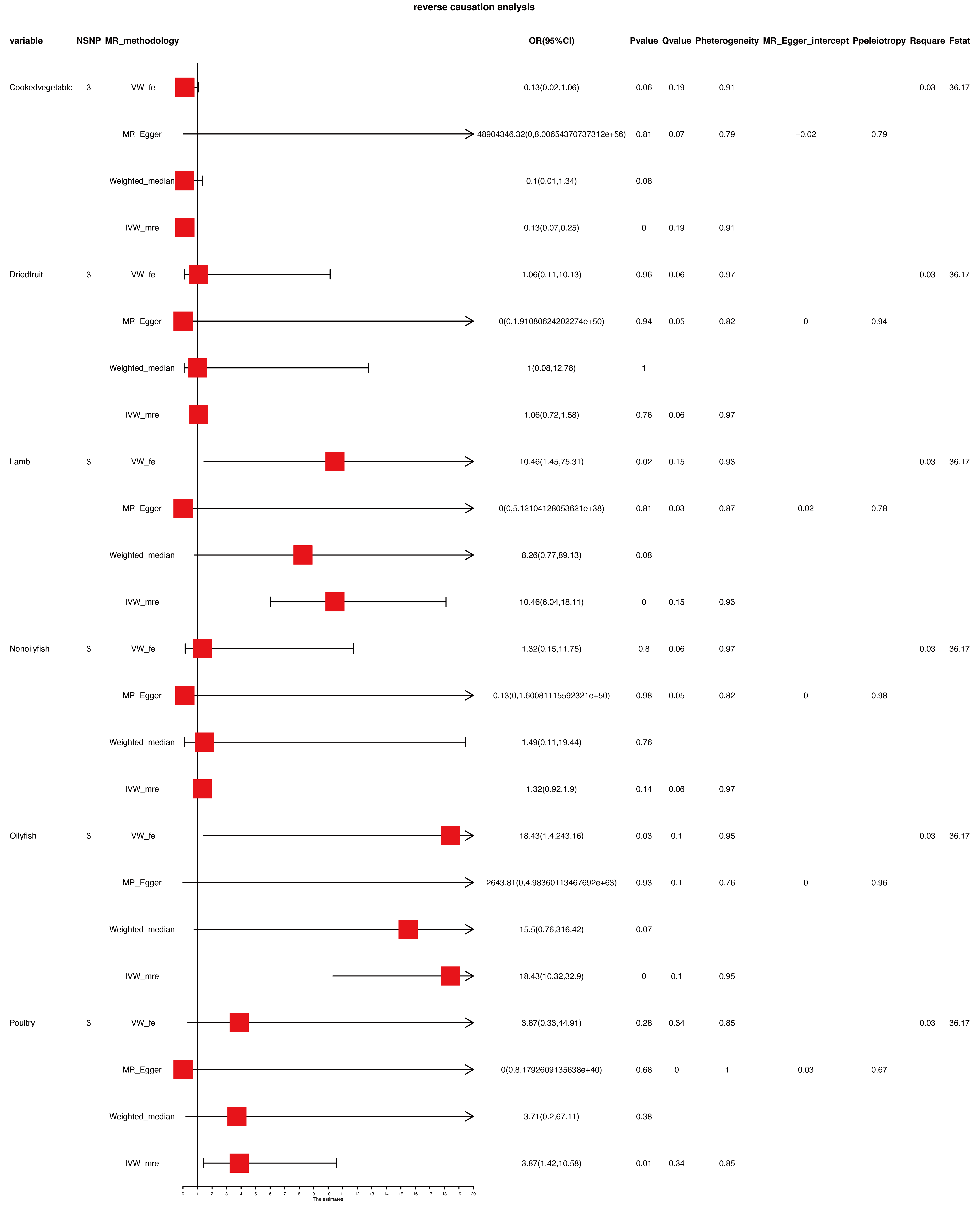

Supplement: Supplymentary Figure S1 — Reverse causation analysis. [file Image_1.TIF]
